# The effect of temperature and heat shock protein 72 on the ex vivo acute inflammatory response in monocytes

**DOI:** 10.1007/s12192-019-00972-6

**Published:** 2019-02-12

**Authors:** Sven P. Hoekstra, Adam K. A. Wright, Nicolette C. Bishop, Christof A. Leicht

**Affiliations:** 1 The Peter Harrison Centre for Disability Sport, Loughborough University, Loughborough, UK; 2 School of Sport, Exercise and Health Sciences, Loughborough University, Loughborough, UK; 3 Department of Infection, Immunity and Inflammation, University of Leicester, Leicester, UK

**Keywords:** Passive heating, Flow cytometry, Inflammation, Interleukin-6, Heat shock protein 72

## Abstract

The acute inflammatory response to active or passive activities that increase body temperature may aid to reduce chronic low-grade inflammation. This study investigates the impact of temperature and extracellular heat shock protein 72 (eHsp72) on the acute intracellular Hsp72 (iHsp72) and interleukin-6 (iIL-6) response in monocytes. Whole blood was incubated for 2 h at 37.0 °C, 38.5 °C and 40.0 °C, in the absence or presence of 0.5 μg/ml eHsp72. Flow cytometry was used to assess iHsp72 and iIL-6 expression in total monocytes and the three monocyte subsets. Incubation at 40.0 °C (*p* < 0.001) but not 38.5 °C (*p =* 0.085) increased iHsp72 expression when compared with 37.0 °C, while there was no effect of temperature on iIL-6 expression (*p* = 0.635). Following incubation with eHsp72, the expression of iHsp72 in classical monocytes was reduced at all temperatures (*p* < 0.001), while there was no effect of eHsp72 on iIL-6 expression (*p* = 0.071). Large temperature elevations are needed to induce an acute iHsp72 response in monocytes. In addition, contrary to its suggested role as a danger signal for the innate immune system, eHsp72 reduced iHsp72 and iIL-6 expression in monocytes.

**Electronic supplementary material:**

The online version of this article (10.1007/s12192-019-00972-6) contains supplementary material, which is available to authorized users.

## Introduction

The exercise-induced acute inflammatory response is suggested to partly mediate the reduction in chronic low-grade inflammation following chronic exercise training (Petersen and Pedersen 2005). The acute response following exercise is duration and intensity dependent (Fischer 2006), further influenced by exercise-related stressors such as oxidative stress, hypoxia and glycogen depletion (Fischer 2006; Noble et al. 2008). The rise in body temperature is an additional determinant of its magnitude. Clamping of core temperature (*T*_core_) during exercise, for instance, dampens (Laing et al. 2007) or in some cases even completely abolishes the acute inflammatory response (Mestre-Alfaro et al. 2012; Rhind et al. 2004).

The acute inflammatory response to active (i.e. exercise) and passive body temperature elevations (e.g., hot water immersion (HWI), sauna therapy) is characterised by the production of cytokines and other proteins, such as interleukin (IL)-6 but also heat shock protein 72 (Hsp72). The latter protein exerts a range of functions, dependent on its location (Noble et al. 2008). While intracellular heat shock protein (iHsp72) mainly functions as a chaperone for correct protein folding and protects against cellular damage in response to physiological stress (Noble et al. 2008), extracellular heat shock protein (eHsp72) can activate monocytes in an lipopolysaccharide (LPS)-like manner by binding to Toll-like receptor (TLR)2 and TLR4 in conjunction with cluster of differentiation (CD)14 (Asea et al. 2000; Johnson and Fleshner 2006). While the increased expression of these inflammatory markers following physiological stress is manifested in a variety of tissues (e.g., skeletal muscle and adipose tissue), its assessment in monocytes provides a less intrusive method to investigate such responses. Moreover, in comparison to other leukocyte subtypes, monocytes are particularly responsive to heat stress (Oehler et al. 2001). Irrespective of the tissue assessed, iHsp72 is increasingly recognised for its beneficial effects on insulin signalling, potentially via attenuating the activity of pro-inflammatory pathways such as those regulated by c-Jun N-terminal kinase (JNK) and nuclear factor-κB (NF-κB) (Henstridge et al. 2014). Thus, inducing an acute inflammatory response and elevating iHsp72 expression in monocytes using heat stress may form an alternative approach to improve metabolic health. Additionally, as monocytes are increasingly recognised for their influence on cardiovascular health (Heine et al. 2012), adaptations in this leukocyte subtype may aid to reduce chronic low-grade inflammation (Devaraj et al. 2008; Flynn and McFarlin 2006; Henstridge et al. 2014).

Although monocytes have been shown responsive to heat stress (Oehler et al. 2001), the thermal stress needed to increase their expression of iHsp72 and IL-6 (iIL-6) is yet unknown. Immersion of humans in water set at 39.5 °C for 2 h, for instance, induces an acute iHsp72 response (Oehler et al. 2001). However, a more modest thermal load (i.e. 1 h HWI in water set at 39 °C) does not result in an increased iHsp72 expression (Morton et al. 2007; Hoekstra et al. 2018), although iHsp72 mRNA expression is elevated after 30 min of sauna bathing at 98.2 °C and 10% humidity (Zychowska et al. 2017). While a temperature threshold may therefore exist for iHsp72, the effect of heat on iIL-6 is less clear. Starkie et al. (2005) found no effect of additional heat stress during exercise on monocyte iIL-6 expression, and in vitro incubation of monocytes at elevated temperatures can actually reduce iIL-6 production by macrophages (Fairchild et al. 2000). It should be noted that previous studies have assessed the acute inflammatory response in total monocytes only (Oehler et al. 2001; Starkie et al. 2005). However, monocytes consist of three subsets (i.e. classical, intermediate and non-classical monocytes), differing in their expression and production of inflammatory markers in response to stress (Mukherjee et al. 2015). It is not known whether these subsets also differ in their acute inflammatory response to temperature elevations.

Therefore, the aims of this study are twofold: to investigate (1) the influence of temperature elevations on the acute iIL-6 and iHsp72 response in monocytes and monocyte subsets, and (2) whether increased concentrations of eHsp72 mediate the acute inflammatory response in monocytes and monocyte subsets in response to elevated temperatures. Together, this can inform interventions that manipulate body temperature using passive (e.g. HWI, sauna therapy) or active methods (i.e. exercise) to induce changes in the inflammatory profile of monocytes.

## Methods

### Procedures

Twelve healthy, recreationally active males (age 29.8 ± 4.2 years; body mass index 25.7 ± 5.7 kg/m^2^; structured exercise 4.0 ± 4.2 h/week) visited the laboratory following an overnight fast. Participants refrained from exercise, caffeine and alcohol on the day preceding the laboratory visit. After providing informed consent, participants rested in a seated position for 15 min. Thereafter, blood was drawn from an antecubital vein into a K_3_EDTA tube. Six 1 ml aliquots of whole blood were pipetted into separate Eppendorf tubes. Whole blood was chosen to reduce the possible effects of separation techniques, while at the same time using a physiologically relevant model to study monocyte function (Mukherjee et al. 2015). The tubes were incubated for 2 h at 37.0 °C, 38.5 °C or 40.0 °C using heat blocks, in the presence or absence of 0.5 μg/ml recombinant low endotoxin eHsp72 (Enzo life sciences, Farmingdale, USA). These temperatures were chosen to cover the complete range of *T*_core_ generally observed in exercise and passive heating studies (Gibson et al. 2016; Oehler et al. 2001). Temperatures exceeding 40 °C were not deemed applicable to health-promoting strategies in non-athletic populations for safety and motivational reasons. The latter is supported by the reported feelings of discomfort during passive heating (resulting in a peak *T*_core_ of ~ 38.7 °C), possibly interfering with adherence to such interventions (Hoekstra et al. 2018). Polymyxin-B 10 μg/ml (Enzo life sciences) and Brefeldin-A 5 μl/ml (Biolegend, San Diego, USA) were added to all tubes. The temperature of each heat block was checked throughout using a calibrated thermometer, and the tubes were gently inverted every 30 min during the incubation period.

### Flow cytometry

Immediately following incubation, 60 μl whole blood of each condition was added to 5 μl CD14, 2.5 μl CD16, 2 μl CD56 antibodies and 2 μl Fc-block (Miltenyi Biotec, Teterow, Germany). After 15 min incubation at room temperature in the dark, 750 μl lysing solution (BD Biosciences, San Diego, US) was added, and cells were incubated for another 10 min. Thereafter, cells were washed with 1.5 ml phosphate-buffered saline (PBS). After fixation and permeabilisation of the cells using Leucoperm (BD Biosciences), 4 μl Hsp72 antibody, isotype control or 2 μl IL-6 antibody were added. After 30 min incubation at room temperature in the dark, cells were washed with PBS, resuspended in PBS and run through the flow cytometer (FACS Calibur, BD Biosciences). The total time elapsed from the start of the staining protocol until running of the samples was 2 h. All antibodies except for IL-6 (Thermo Fisher Scientific, Rockford, IL, USA) were purchased from Miltenyi Biotec.

Cell Quest software (BD Biosciences) was used for the analysis of the 100,000 events collected for each condition. Following exclusion of CD56 positive natural killer cells, monocytes were selected based on positive CD14 expression (Ziegler-Heitbrock et al. 2010). The monocyte subset distribution (CD14++CD16-classical monocytes, CD14++CD16+ intermediate monocytes and CD14+CD16++ non-classical monocytes) was determined using the trapezoid method (Zawada et al. 2015). The iHsp72 and IL-6 expression in total monocytes and monocyte subsets were determined by subtracting the isotype control geometric mean fluorescence intensity (GMFI) from the GMFI of iHsp72 and iIL-6, respectively (see Fig. S1 (supplementary material) for an illustration of the gating strategy used).

### Statistical analyses

Outcomes are given in mean ± SD. Normality of the data was checked using the Shapiro-Wilk test. A Greenhouse-Geisser correction was applied when the assumption of sphericity was violated, which was tested with Mauchly’s sphericity test. Repeated measures ANOVAs were conducted for all analyses, with a Bonferroni correction to test the effect of temperature and eHsp72 in the 4 related cell types (i.e. total, classical, intermediate, and non-classical monocytes). Significant main or interaction effects were followed up by Bonferroni adjusted post hoc paired *t* tests. Due to problems with monocyte subset identification for two participants, *N* = 12 for total monocytes and *N* = 10 for the monocyte subsets. The 23rd version of SPSS (Chicago, USA) was used for all analyses.

## Results

**Fig. 1:**
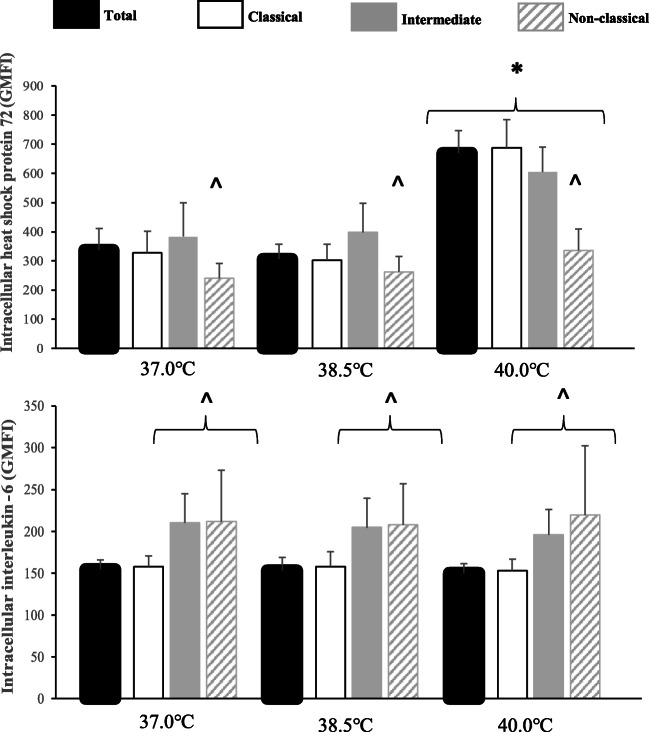
The expression of iHsp72 and iIL-6 in total monocytes and monocyte subsets following incubation of whole blood for 2 h at 37.0 °C, 38.5 °C and 40 °C in the presence of Polymyxin-B and Brefeldin-A. Protein expression was assessed by flow cytometry. Significantly different from (*) other temperatures and (^) other monocyte subsets. *N* = 12 for total monocytes; *N* = 10 for monocyte subsets

In the control condition (incubation at 37.0 °C in the absence of eHsp72), iHsp72 expression in non-classical monocytes was lower when compared with intermediate and classical monocytes (*p* = 0.009). Intracellular IL-6 expression was higher in intermediate and non-classical compared with classical monocytes (*p* = 0.018) (Fig. 1). The monocyte subset distribution following the control condition was as follows: classical monocytes 92.6 ± 2.3%, intermediate monocytes 1.28 ± 0.88%, non-classical monocytes 2.46 ± 0.94%. The proportions of classical (*p* = 0.240), intermediate (*p* = 0.174) or non-classical monocytes (*p* = 0.232) were not affected by temperature elevation, nor incubation with eHsp72 (classical monocytes *p* = 0.293; intermediate monocytes *p* = 0.176; non-classical monocytes *p* = 0.233).

### The effect of temperature elevations on the acute inflammatory response

Incubation at 40.0 °C resulted in an increased iHsp72 expression in total (*p* < 0.001), classical (*p* < 0.001), intermediate (*p* < 0.001) and non-classical monocytes (*p* = 0.002), compared with incubation at 37.0 °C and 38.5 °C (Fig. 1). There was a temperature × monocyte subset interaction, with a lower iHsp72 expression in non-classical monocytes following incubation at 40.0 °C compared with the other monocyte subsets (*p* < 0.001). There was no difference in iHsp72 expression for total monocytes or monocyte subsets between 37.0 °C and 38.5 °C (*p* > 0.085). There was no effect of temperature on the iIL-6 expression in total monocytes (*p* = 0.635) or any of the monocyte subsets (*p* > 0.412).

### The effect of eHsp72 on the acute inflammatory response

**Fig. 2:**
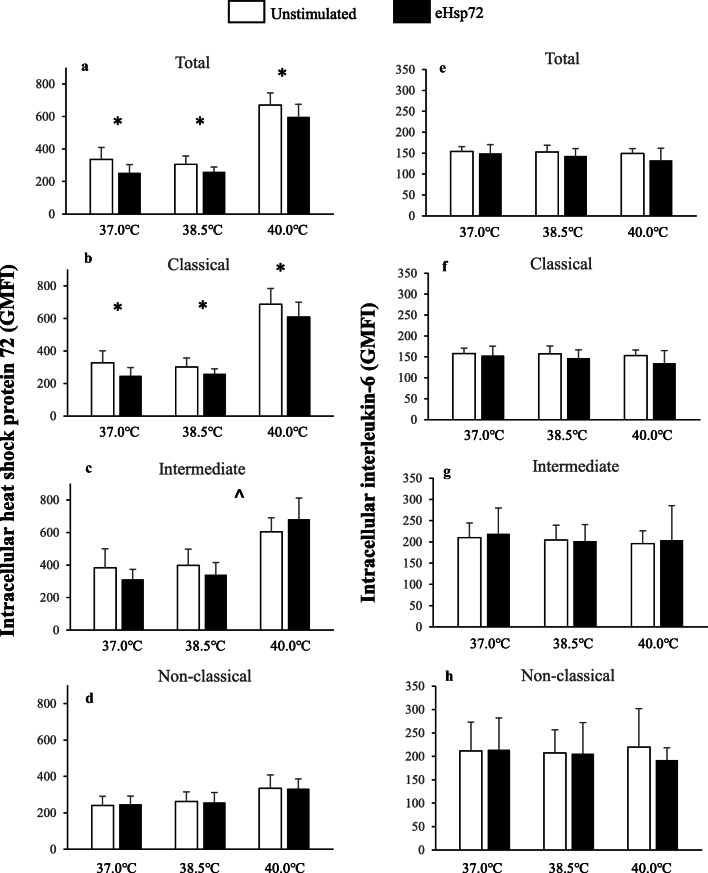
The effect of incubation with eHsp72 on iHsp72 and iIL-6 expression in total monocytes and monocyte subsets at 37.0 °C, 38.5 °C and 40 °C. Whole blood was incubated for 2 h in the presence or absence of 0.5 μg/ml eHsp72. Polymyxin-B and Brefeldin-A were added to each tube. Protein expression was assessed by flow cytometry. Panel **a**–**d** illustrate the iHsp72 expression in total monocytes and the monocyte subsets, panel e-h the iIL-6 expression in the same cell types. * Significant effect of eHsp72, ^ significant Temperature × Condition effect. *N* = 12 for total monocytes; *N* = 10 for monocyte subsets

Incubation with eHsp72 resulted in a lower iHsp72 expression in total monocytes at all temperatures (*p* < 0.001) (Fig. 2a). The same effect was present in classical monocytes (*p* < 0.001), but not in intermediate (*p* = 0.436) and non-classical monocytes (*p* = 0.920) (Fig. 2b–d). There was no change in iIL-6 expression in total monocytes (*p* = 0.074) or any of the monocyte subsets (*p* > 0.074) following incubation with eHsp72 (Fig. 2e–h).

### The interaction between temperature elevation and eHsp72

There was a significant Temperature × Condition interaction for iHsp72 expression in intermediate monocytes, where the presence of eHsp72 resulted in a larger increase in iHsp72 expression between 38.5 °C and 40.0 °C when compared with incubation in the absence of eHsp72 (*p* = 0.004) (Fig. 2c). No interaction effects were present for iHsp72 expression in total, classical or non-classical monocytes (*p* > 0.117). There was also no Temperature × Condition interaction for iIL-6 expression in total monocytes (*p* = 0.646) or any of the monocyte subsets (*p* > 0.641).

## Discussion

As incubation of whole blood at 40.0 °C but not 38.5 °C elevates iHsp72 expression, the results of the present study indicate that large temperature elevations are needed to increase the expression of this intracellular chaperone in monocytes. In addition, despite being referred to as a “danger signal” (Johnson and Fleshner 2006), incubation of whole blood with eHsp72 resulted in a significant reduction in iHsp72. Finally, differential acute responses to heat as well as eHsp72 were observed among the three monocyte subsets, stressing the importance of distinguishing between the subsets when assessing acute responses to potential health-promoting strategies.

The observation that large temperature elevations are needed to elevate iHsp72 expression is in line with previous exercise and passive heating studies. Morton et al. (2007) and Hoekstra et al. (2018) showed that HWI for 60 min, resulting in a peak *T*_core_ of 38.9 ± 0.2 °C and 38.7 ± 0.4 °C, respectively, fails to increase iHsp72 expression. On the other hand, Oehler et al. (2001) observed a significant increase in iHsp72 expression in monocytes following 2 h incubation of whole blood at 39 °C, suggesting that a temperature threshold close to 39.0 °C to increase iHsp72 production may exist. Supporting this notion, Gibson et al. (2016) reported that a *T*_core_ higher than 38.5 °C needs to be maintained for at least 27 min to induce the transcription of Hsp72 mRNA in leukocytes following exercise. Ultimately, the need for such large *T*_core_ increases may hinder the feasibility of interventions aimed to increase iHsp72 expression in monocytes to improve the resting inflammatory profile and metabolic health. It is noteworthy, however, that iHsp72 expression in monocytes constitutes only part of the inflammatory profile and that improvements in metabolic health markers following a chronic passive heating intervention are reported in the absence of acute changes in its expression (Hoekstra et al. 2018).

The absence of an acute iIL-6 response following heat stress is in line with other in-vitro studies, in which phagocytic capacity (Roberts and Steigbigel 1977) and iIL-6 production of monocytes (Fairchild et al. 2000) were not affected by elevated temperatures. Moreover, additional heat stress during exercise does not alter monocyte iIL-6 production in response to LPS stimulation (Starkie et al. 2005). Therefore, it is conceivable that the increased plasma IL-6 concentrations following in vivo passive heating reported in previous studies (Faulkner et al. 2017; Laing et al. 2007; Leicht et al. 2015) originated from tissues or organs other than monocytes (e.g. skeletal muscle).

Incubation of whole blood with eHsp72 reduced the expression of iHsp72 in total and classical monocytes at all three temperatures. Moreover, although this did not reach statistical significance, the same trend was found for iIL-6. This contrasts with the suggestion that eHsp72 may serve to amplify the inflammatory response to heat stress by acting through the same mechanisms as a damage-associated molecular pattern after its secretion into the circulation (Gupta et al. 2010). While LPS indeed amplifies iHsp72 production during heat stress (Gupta et al. 2010), it may be that eHsp72 does not activate immune cells in an LPS-like manner in all situations (Van Eden et al. 2012). Indeed, although incubation with eHsp72 can induce cytokine production in monocytes and macrophages (Asea et al. 2000; Campisi et al. 2003; Galdiero et al. 1997), support for a mere anti-inflammatory role of eHsp72 exists. For instance, Ferat-Osario et al. (2014) showed that eHsp72 can downregulate the acute inflammatory response to stimulation with TLR agonists in peripheral mononuclear blood cells, while Hulina et al. (2018) observed similar anti-inflammatory actions of eHsp72 when cells of a monocytic cell line were incubated with a TLR2 agonist. Although the conflicting literature on the role of eHsp72 in the acute inflammatory response (Ferat-Osario et al. 2014; Asea et al. 2000; Van Eden et al. 2012) makes it difficult to make firm suggestions on the mechanisms underlying the reduced iHsp72 expression following incubation with eHsp72 observed in the present study, the potential absorption of exogenous Hsp72 by the cells may form part of an explanation. The absorbed exogenous Hsp72 can form a complex with heat shock factor-1 (HSF-1), reducing the activity of the latter (Abbravaya et al. 1992). As binding of HSF-1 with elements on the heat shock gene can induce both iHsp72 and iIL-6 production (Welc et al. 2013), the formation of these complexes may reduce the expression of both proteins. Of note, the reduction in iHsp72 expression was not present in intermediate and non-classical monocytes. As TLR stimulation by eHsp72 is CD14 dependent (Asea et al. 2000), the lower CD14 expression on non-classical monocytes may provide a lead to yet another potential mechanism by which eHsp72 can reduce the expression of inflammatory markers in monocytes (Cros et al. 2010).

Some limitations of this study need mentioning. First, the acute inflammatory response to heat may be influenced by age (Njemini et al. 2002) and physical fitness (Selkrik et al. 2009). Therefore, as only recreationally active, healthy young males were included in the present study, caution needs to be applied when translating the findings to other populations. Furthermore, in investigating the impact of temperature elevations on inflammatory markers in the context of chronic low-grade inflammation, the assessment of anti-inflammatory cytokines such as IL-10 and IL-1ra would have been a useful additional outcome variable. However, due to the limited number of parameters that can be analysed with the flow cytometer available this was not an option in the present study.

In summary, this study showed that incubation of whole blood at 40.0 °C but not 38.5 °C elevates iHsp72 expression in monocytes. Furthermore, incubation with eHsp72 reduced iHsp72 expression in monocytes; which is in contrast with its suggested role as a danger signal for the innate immune system. The potential practical implications of these results are that strategies aiming to increase iHsp72 expression in monocytes by repeated passive or active heating to improve cardiometabolic health may need to induce large elevations in body temperature to do so.

## Electronic supplementary material

Fig S1 Gating strategy used to assess iHsp72 and iIL-6 expression in total monocytes and monocyte subsets. Following exclusion of CD56+ natural killer cells, monocytes were selected based on their CD14+ expression. Thereafter, monocyte subsets were determined based on CD14 and CD16 expression, after which the iHsp72 and iIL-6 expression was assessed in the total monocytes as well as the monocyte subsets. (PDF 151 kb)
