# Supplementary figures and images for: The effect of temperature and heat shock protein 72 on the ex vivo acute inflammatory response in monocytes
Source: Cell Stress Chaperones. 2019 Feb 12;24(2):461–7. doi: 10.1007/s12192-019-00972-6 (PMC6439050; doi:10.1007/s12192-019-00972-6)

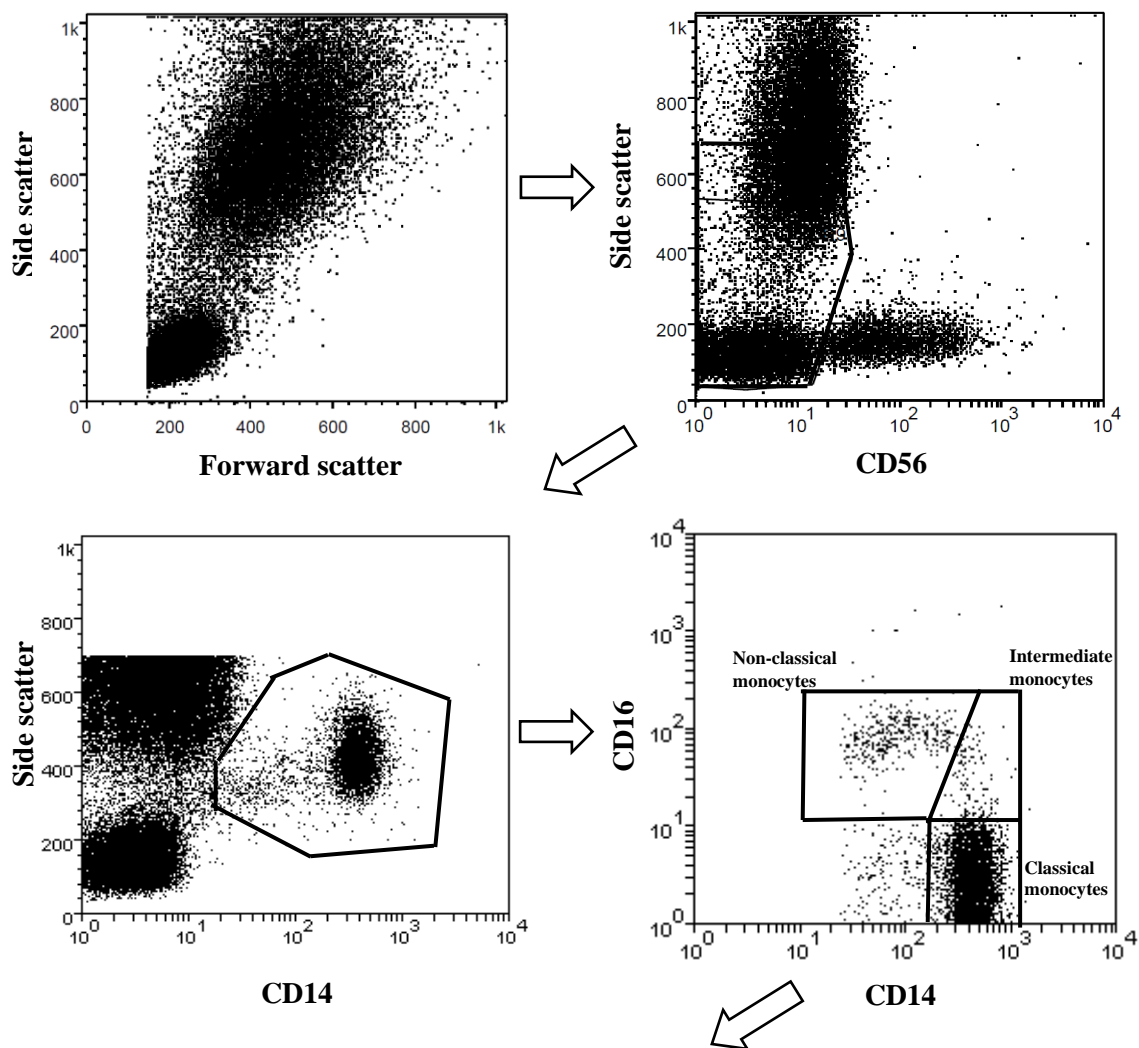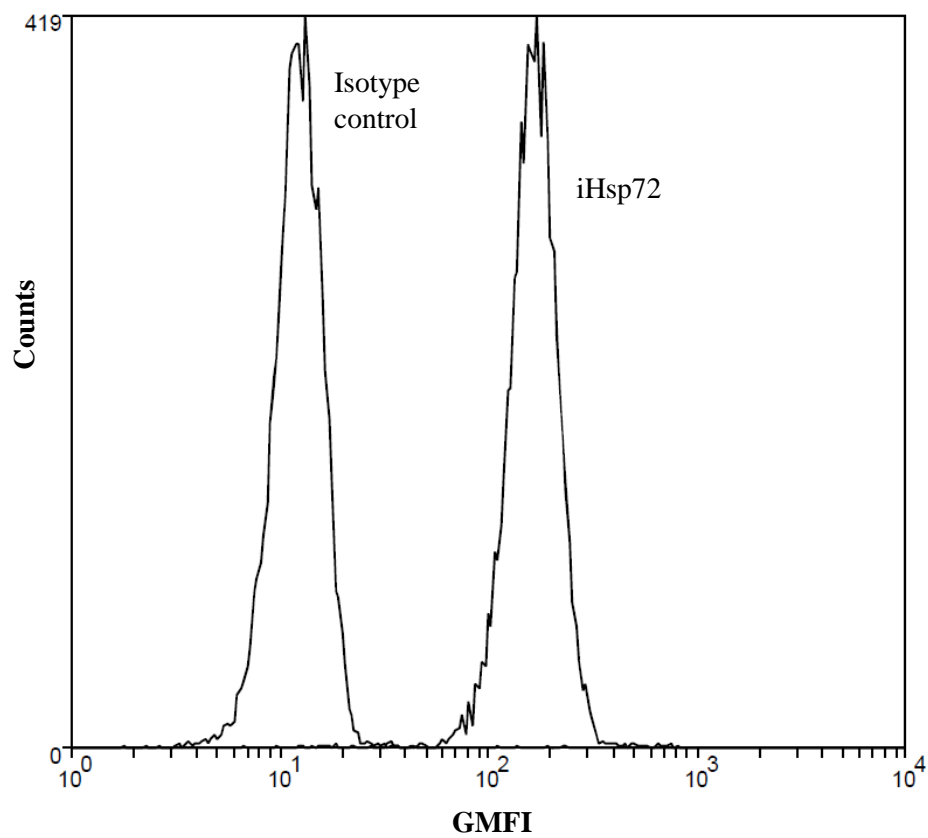

Supplement: Supplementary file 1 — Gating strategy used to assess iHsp72 and iIL-6 expression in total monocytes and monocyte subsets. Following exclusion of CD56+ natural killer cells, monocytes were selected based on their CD14+ expression. Thereafter, monocyte subsets were determined based on CD14 and CD16 expression, after which the iHsp72 and iIL-6 expression was assessed in the total monocytes as well as the monocyte subsets. (PDF 151 kb) [file 12192_2019_972_MOESM1_ESM.pdf]
